# Microbial Musings – August 2020

**DOI:** 10.1099/mic.0.000969

**Published:** 2020-08-26

**Authors:** Gavin H. Thomas

**Affiliations:** ^1^​ Department of Biology, University of York, York, UK

With many of us are taking some form of break in August to recharge our batteries for the oncoming academic year, there is no better time to flick through the latest issue of *Microbiology* to find an article that gives you some broader background on a subject or introduces you to something totally new. The first article in this issue aims to do just that and is our first early-career researcher (ECR) topic review. The journal has been working over the last 18 months with a group of ECRs who all work on different aspects of the biology of actinomycetes, mainly of the genus *Streptomyces *. Having first met with them at the Microbiology Society annual conference in Belfast in 2019, the journal sponsored their development of Actinobase (actinobase.org), a Wiki-based community repository for all things actinomycete, which they actively curate by adding a large variety of content for microbiologists around the world. We proposed as a way to further connect the journal to its community that they write, as a group of ECRs, an annual topic review for the journal highlighting their personal favourite papers in actinomycete research from 2019 [1]. The authors, Sam Prudence (@Sam_Prudence), Emily Addinton (@emyaddington), Laia Castaño-Espriu (@Laia_Castano), David Mark (@DavidRcoMark), Linamaria Pintor-Escabar (@LinaPintorE), Alicia Russell (@alisia_russell) and Tom McLean (@TomMcLean05), bring together papers across four major areas of actinomycete research from development and regulation, specialized metabolite production, ecology and host interactions and technologies and methodology. They pick and describe their ten favourite papers in these areas from 2019 – a must for actinomycete aficionados and for all microbiologists seeing how work in this model antibiotic-producing species is providing fundamental insights into microbial processes. I hope that this is not just the first one of these reviews on actinomycetes, but as we seek to form new ECR-led communities in 2021 then more groups of young researchers can come together to bring their unique perspective on what is hot in their field.

Our first research paper is from an Actinobacterium, namely *Corynebacterium glutamicum *, and concerns the mechanisms of localization of lipoproteins in the cell envelope. The maturation of these proteins, secreted first through the inner membrane by Sec or Tat pathways, is pretty similar in most bacteria and an enzyme, Lgt, recognizes a conserved cysteine residue just beyond the N-terminal signal peptide to attach a lipid anchor to the protein. The signal peptide is then removed by a specific signal peptidase (LspA) and the mature protein wanders around the surface of the inner membrane to undertake its function, where is can be further acylated by another protein Lnt. In Gram-negative bacteria there are additional processes that direct a subset of these proteins to the outer membrane [2, 3]. Due to a lack of a classical periplasm in Gram-positive organisms, substrate binding protein (SBP)-dependent transporters have to anchor the SBP to the inner membrane and are usually lipoproteins. In this work from Nathalie Dautin and colleagues at the Université Paris‐Saclay, France, the authors have looked at the functions of Lgt and LspA in *C. glutamicum* [4]. Previous work in the related Actinobacterium *Streptomyces coelicolor,* which has two Lgts, suggested that this function was essential as a double mutant could not be constructed [3] and in the related *S. scabies* the *lgt* mutant had a severe growth phenotype [5]. However, in *C. glutamicum* while Lgt is required for acylation and membrane anchoring, it is not essential for growth. Interestingly, they see that the lipoprotein MusE, which is the SBP of a maltose ABC transporter in this bacterium, is now secreted in the *lgt* mutant. It is moved across the inner membrane normally by the Sec complex, but not anchored and floats away, which is similar to phenotypes seen in *lgt* mutants of *Mycobacterium smegmatis* [6]. Fascinatingly they show that while MusE is essential for maltose uptake and growth on maltose as the sole source of carbon [7], its ‘released’ soluble version could still function to some degree without being anchored to the membrane. It is known that *C. glutamicum* has an atypical outer membrane, dubbed the ‘mycomembrane’ and perhaps this retains sufficient soluble MusE to continue efficient maltose transport. The authors also show that the acylation is not required for subsequent *O*-linked glycosylation of lipoproteins, hence these two post-translational modifications are not functionally coupled. Certainly, we have lots more to learn about the structure, function and biogenesis of the envelope of these interesting bacteria.

In this issue we have multiple papers on various aspects of biofilms and we start with a paper on the bacterium *Pseudomonas fluorescens* SBW25, famous not just as a rhizosphere colonizer but also as it can evolve multiple types of biofilm forms, using cellulose or poly-acetyl glucosamine (PGA) as the matrix. In a paper in this issue from the group of Andrew Spiers, at Abertay University, UK, the authors compare the properties of a number of strains derived from SBW25 in different ways that have evolved different biofilm types to grow at the air–liquid (A–L) interface [8]. This includes the classical wrinkly spreader (WS) biofilm type, discovered by Paul Rainey and Michael Travisano (@mtravisano), when at the University of Oxford, UK, that evolved at the A–L interface in liquid cultures in unstirred beakers from the planktonic growing parent strain [9]. The WS strains have mutations that increase production of an acetylated cellulose biofilm matrix that allows them, as a population, to effectively colonize this A–L interface [10] and this is a system that still serves as a model for the evolutionary routes to adaptive phenotypes [11]. Here the authors show that their three different A–L biofilm types have different properties in terms of strength, adhesiveness and rheology, and when they compete them directly against each other to colonize the A–L interface from 1 : 1 mixed inoculum they find that the WS type is actually poorly competitive to the other types, although this also varies depending on the ratios of the two types added at the start. Overall, the paper illustrates how different types of biofilm characteristics can emerge from a single ancestral-strain, which can confer different fitness advantages under different conditions, highlighting the adaptive variability of environmental bacteria.

Another paper in this issue looks at an aspect of the complex regulation of biofilm formation in the related Pseudomonad, *Pseudomonas aeruginosa *. The paper, from the group of Cristina Costa and colleagues from the Comisión Nacional de Energía Atómica, Argentina, examines the mechanism by which solar UV irradiation (UVA of 315–400 nm wavelength) induces biofilm formation and finds key known biofilm formation genes are induced [12]. By demonstrating the essentiality of *relA* for UVA induction of biofilm formation they propose a model whereby UVA stress induces RelA function and ppGpp production, which is a known inducer of biofilm formation through the activation of various quorum-sensing mechanisms. They hypothesize that the UVA can alter the aminoacylation capacity of certain tRNA, hence triggering RelA and ppGpp production, like in the stringent response. In fact a similar mechanism has been known in *Escherichia coli* for many years where UVA induces low levels of acylation of particular tRNAs [13]. While not entirely ruling out the role of reactive-oxygen-species (ROS) formation in the process they suspect that the RelA-mediated response is the primary one.

The paper that has been chosen by senior editor Ricardo Manganelli as this month’s editor’s choice focusses on a related area in the biology of *P. aeruginosa *, namely the evolution of cheaters in populations of this bacterium [14]. The paper from James Gurney (@Jamesrgurney) working in the lab of Steve Diggle (@DiggleLab) with Sam Brown (@sambrownlab) at Georgia Institute of Technology, USA, tests ideas around the evolution of cheaters, which can arise readily by mutating genes that produce quorum-controlled factors, which gives the mutants a fitness advantage [15]. In this work, which is described by Ricardo in the Microbiology Society blog, the authors look at combinatorial quorum sensing, whereby the cells need two different signals to induce quorum sensing, and how this can allow novel cheating strategies to emerge.

Returning to biofilms again, we have a quite different paper about breaking biofilms using amoebae, from the group of Brad Borkee (@BorleeLab) at Colorado State University, USA [16]. In this work the authors look at cell-free supernatants from a variety of different predatory amoebae, which can naturally eat bacteria living in preformed biofilms, and look at the ability of these extracts to actively break down the biofilm matrix. Interestingly they find that not only do the extracts actively reduce biofilm mass, presumably due to active dispersion, but in one case they also actively kill planktonic cells of methicillin-resistant *Staphylococcus aureus* (MRSA). The precise dispersion mechanism is not known, although the extracts are known to contain various hydrolytic enzymes that could digest the matrix polymers and the dispersion activity is lost when the extracts are heat treated, suggesting an active enzymatic component. The authors also hypothesize that the enzymes might be released in extracellular microvesicles (MVs) that could be delivered onto the biofilm surface. A very cool area for future research to exploit these bacteria-eaters.

The concept of secreted vesicles leads us very nicely to the next paper that caught my eye, which is on the protective effects of outer membrane vesicles (OMVs) produced by *Helicobacter pylori *. In this paper from the group of Jody Anne Winter (@WinterJody) and colleagues at Nottingham Trent University, UK, the authors look for additional functions of OMVs in the biology of this gastric pathogen. The released OMVs are known to contain outer membrane proteins and periplasmic constituents and can deliver virulence factors into host cells when they are taken up [17]. Here they show dose-dependent protection against hydrogen peroxide and certain antibiotics including clarithromycin, but not others such as amoxicillin. The enhanced protection against hydrogen peroxide by OMVs has been observed recently in a different strain of *H. pylori* and shown to be dependent on catalase activity of the OMVs [18]. However, the protection against clarithromycin is novel and the mechanism is unclear; it could be simple sequestration of this hydrophobic compound by the OMVs, although the same protection is not seen for other similarly hydrophobic antibiotics, or a particular protein component of the OMVs might bind the antibiotic. Intriguingly they find that OMVs appear to be able to interact with the antimicrobial peptide LL-37, which results in increased growth of *H. pylori* at high concentrations of LL-37 compared to low concentrations. Perhaps the vesicles have some protease activity that helps them to digest the LL-37 into shorter peptides that can be transported into *H. pylori* and used as a nutrient and in fact at least one protease, HtrA, is already known to be enriched in *H. pylori* OMVs [19].

The final article that caught my attention in this issue considers natural reservoirs for antimicrobial resistance genes by looking at a commensal species of Staphylococcus found in the oral microbiota of domestic cats [20]. In the study from the group of Marcia Giambiagi-deMarval at the Universidade Federal do Rio de Janeiro, Brazil, they examine how *Staphylococcus nepalensis* harbours resistance genes for many antibiotics located on plasmids that can be easily conjugated into *S. aureus *, building on her earlier work more generally on the potential of diverse commensal Staphylococci to be reservoirs of antimicrobial resistance genes [21]. This could extend to human skin itself, which is known to harbour many diverse species of Staphylococci, many of which are not pathogens per se [22, 23].

We will be looking for new editors and senior editors for the journal this coming month so look out for the adverts if you are interested and for ECRs who wish to get involved in the journal then please look out for the advert to join to our Board of Reviewers to get your first opportunity of peer reviewing and helping to #publishforthecommunity.
